# Immunoglobulin G4-related constrictive pericarditis and the importance of a thorough workup: a case report

**DOI:** 10.1186/s12872-022-02468-1

**Published:** 2022-02-04

**Authors:** Sérgio Maltês, Margarida Cabral, Pedro Freitas, Catarina Albuquerque, Carolina Fernandes, Diana Moura, Beatriz Santos, Miguel Mendes, José Neves

**Affiliations:** 1 Cardiology Department, Hospital Santa Cruz, Centro Hospitalar Lisboa Ocidental, Av. Prof. Dr. Reinaldo dos Santos, 2790-134 Lisbon, Portugal; 2 Cardiology Department, Hospital Leiria, Leiria, Portugal; 3 Anatomical Pathology Department, Hospital Santa Cruz, Centro Hospitalar Lisboa Ocidental, Lisbon, Portugal; 4 Internal Medicine Department, Hospital Leiria, Leiria, Portugal; 5 Cardiothoracic Surgery Department, Hospital Santa Cruz, Centro Hospitalar Lisboa Ocidental, Lisbon, Portugal

**Keywords:** Case report, Constrictive pericarditis, Immunoglobulin G4-related disease, Right heart failure

## Abstract

**Background:**

Constrictive pericarditis remains a problematic diagnosis and a thorough investigation is critical. Among possible aetiologies, immunoglobulin-G4 (IgG4)-related pericardial disease is an unusual cause of pericardial constriction. We report a challenging diagnostic case of pericardial constriction due to IgG4-related disease.

**Case presentation:**

A 68-year old male with a history of inferior myocardial infarction with right ventricle (RV) involvement was thrice-hospitalized due to marked ascites and peripheral oedema. Systemic congestion was initially attributed to RV dysfunction due to previous infarction. Yet, at the final admission, a re-assessment echocardiogram followed by cardiac computed tomography, magnetic resonance and right heart catheterization raised a possible diagnosis of constrictive pericarditis with a finding of abnormal pulmonary venous return. Patient therefore underwent pericardiectomy and surgical correction of pulmonary venous return. Pericardium histology revealed an IgG4-related pericardial constriction. Patient was later discharged on corticosteroids with marked symptomatic improvement.

**Conclusion:**

IgG4-related disease remains a rare cause of pericardium constriction while also presenting a challenging diagnosis in everyday clinical practice. This case exemplifies the difficulties faced by clinicians when reviewing a possible case of constrictive pericarditis, while highlighting the importance of a multimodality assessment.

**Supplementary Information:**

The online version contains supplementary material available at 10.1186/s12872-022-02468-1.

## Background

ImmunoglobulinG4 (IgG4)-related disease is a systemic syndrome with multiorgan lymphoplasmacytic infiltration. Yet, the frequency of IgG4-related pericarditis remains unknown. Given its non-specific presentation, the correct diagnosis may be delayed, especially in those with other causes of right heart failure (RHF). We present a challenging diagnostic case of constrictive pericarditis due to IgG4-related disease in accordance with the CARE reporting checklist.

## Case presentation

A 68-year old male with a previous inferior myocardial infarction (MI) with right ventricle (RV) involvement was thrice-admitted due to abdominal distension and peripheral oedema. He denied fever, dyspnoea or known hepatic or renal disease. Vital signs were stable and physical examination identified marked ascites and peripheral oedema. Blood tests revealed an NT-proBNP of 1525 pg/mL (reference: < 125 pg/mL), creatinine 1.25 mg/dL (reference: < 0.8–1.3 mg/dL), alanine aminotransferase 30U/L (reference 5-30U/L) and C-reactive protein of 4 mg/dL (reference: < 5 mg/dL) with no proteinuria. Paracentesis unveiled a serum-ascites albumin gradient of 1.3 g/dL with no malignant cells, thus suggestive of portal hypertension.

**Fig. 1:**
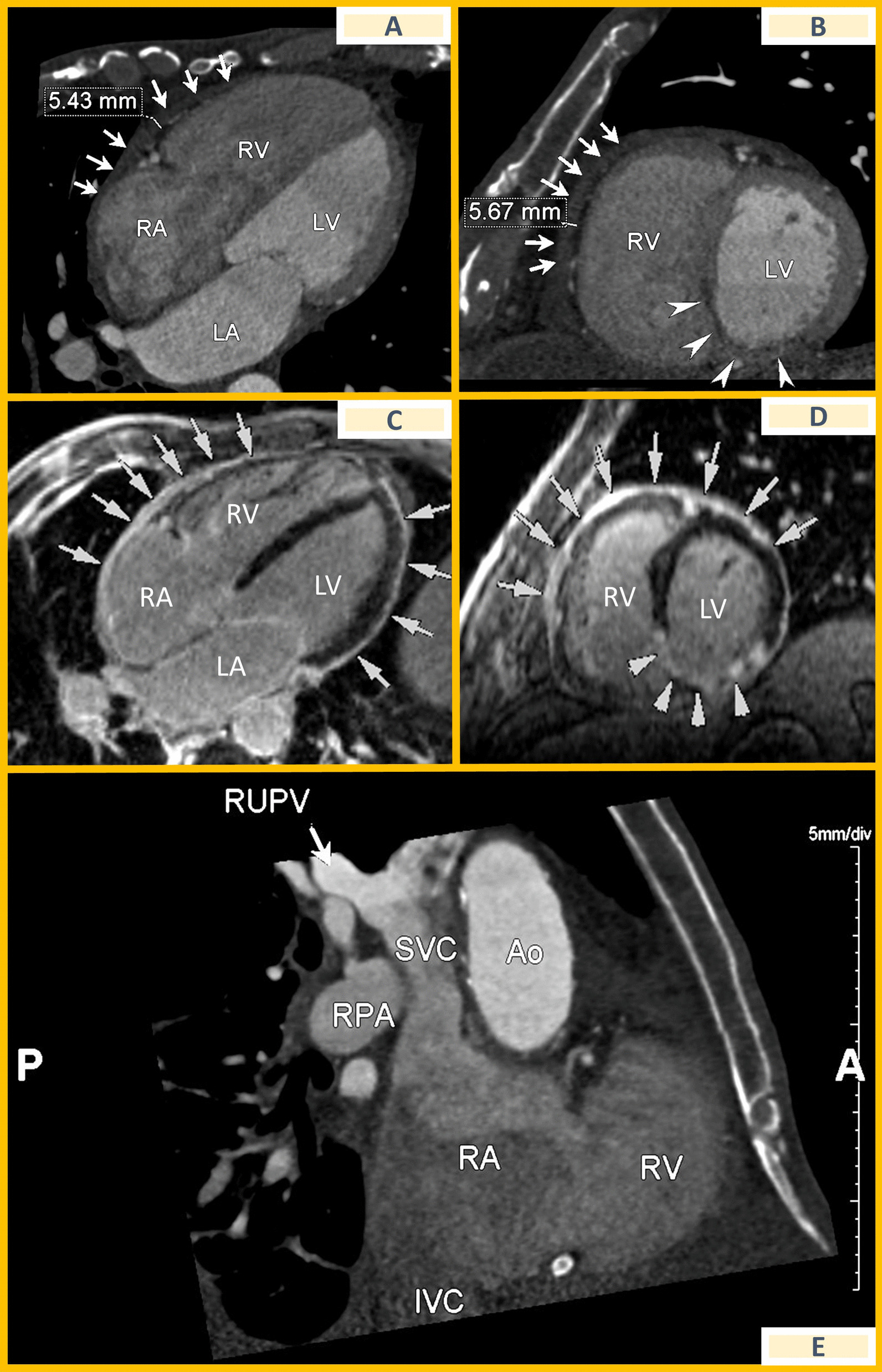
**A** Computed tomography showing thickened pericardium (arrows); **B** computed tomography revealing pericardial thickening (arrows) and inferior myocardial-wall scar (arrowheads); **C** magnetic resonance revealing pericardium late-gadolinium enhancement (arrows); **D** magnetic resonance revealing pericardial thickening (arrows) and inferior myocardial-wall scar (arrowheads); **E** computed tomography showing abnormal pulmonary venous return. Ao = aorta; IVC = inferior vena cava; LA = left atrium; LV = left ventricle; RA = right atrium; RPA = right pulmonary artery; RUPV = right upper pulmonary vein; RV = right ventricle; SVC = superior vena cava

An initial diagnosis of RHF due to previous MI with RV involvement was assumed and intravenous diuretics were started. Yet, a re-evaluation echocardiogram performed during the final hospitalization revealed pericardial thickening, respiration-related ventricular septal shift and a peak septal E' > 8.0 cm/s; however, no obvious variation in mitral inflow E velocity was identified. Echocardiogram also showed dilated inferior vena cava and borderline RV dysfunction (tricuspid annular systolic plane excursion of 16 mm, peak tricuspid annular systolic velocity by tissue-Doppler imaging of 10 cm/s). Cardiac computed tomography (CT) unveiled mild pericardial thickening while also identifying an anomalous pulmonary venous return (right-upper pulmonary vein draining in the superior vena cava) (see Fig. 1A, B, E). Subsequent cardiac magnetic resonance (CMR) confirmed pericardium thickening with diffuse late gadolinium enhancement (LGE) (see Fig. 1C, D and Additional file 1: video 1) and ventricular septum “d-shape”. Estimated Qp/Qs was 1.4. To clarify such findings, a right heart catheterization (RHC) was ordered. RHC revealed a Qp/Qs of 1.7, intracardiac pressure equalization, discordant RV/left ventricle systolic pressure relationship with inspiration and an RV systolic pressure < 50 mmHg with a pressure-waveform showing a “dip and plateau” pattern. A possible diagnosis of constrictive pericarditis with an incidental finding of anomalous pulmonary venous return was considered.

**Fig. 2:**
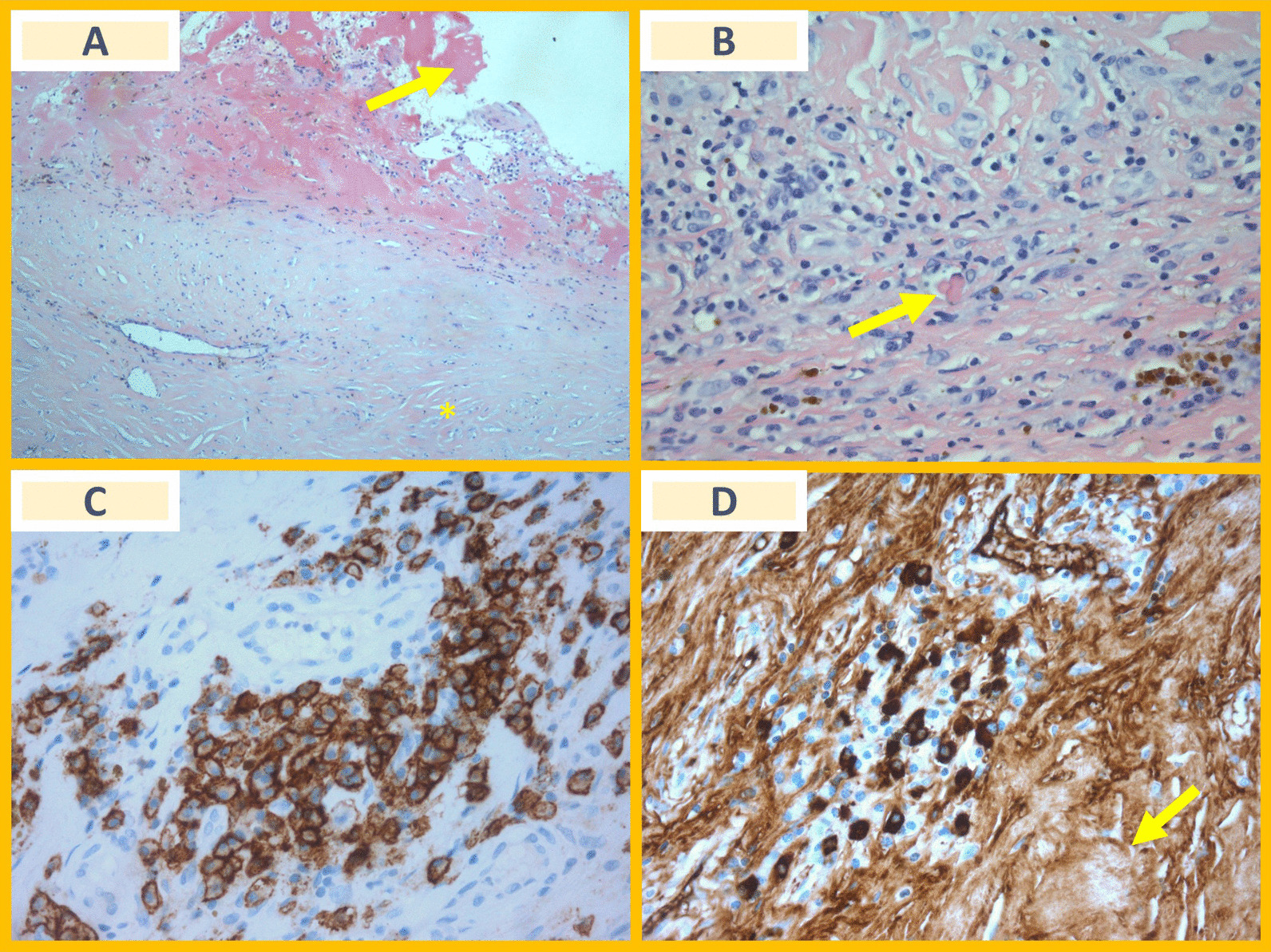
**A** (Hematoxiline-eosin, 100X): Pericardium with diffuse fibrous thickening, with focal areas of intersecting fascicles (storiform pattern—asterisk), and fibrinous areas at the surface (arrow); **B** (Hematoxiline-eosin, 400X): Patchy lymphoplasmacytic infiltration, and areas with Russell bodies (arrow)—atypical plasma cells with eosinophilic, homogeneous immunoglobulin-containing inclusions in cytoplasm; **C** (CD138 immunostaining, 400X): CD138 membranous immunostaining demonstrating plasma cells forming clusters; **D** (IgG4 immunostaining, 400X): cytoplasmatic immunostaining demonstrating an increased absolute count of IgG4-positive cells (> 20 per high-power field) with a marked storiform fibrosis pattern (arrow)

After Heart-Team discussion, patient underwent pericardiectomy and surgical correction of anomalous pulmonary venous return. Post-operative RHC now showed no constrictive physiology. Resected pericardium unveiled diffuse fibrous thickening, as well as a patchy and dense lymphoplasmacytic infiltration with numerous plasmocytes. Moreover, focal fibrosis with a storiform pattern was observed. As such, IgG4 immunochemistry study was performed, which confirmed an extensive IgG4-positive plasma-cell infiltration (Fig. 2A–D).

A diagnosis of IgG4-related constrictive pericarditis with concomitant anomalous pulmonary venous return was therefore established. The patient was started on corticosteroids and discharged with marked symptom improvement.

## Discussion and conclusions

Multimodality imaging is often required in diagnosing constrictive pericarditis. Several concurring RHF aetiologies were presented during this patient’s work-up, namely: (a) previous MI with RV involvement; (b) cardiac CT with pericardial thickening and anomalous pulmonary venous return also contributing to RV volume overload and restrictive physiology; (c) CMR revealing diffuse pericardial LGE and a Qp/Qs of 1.4. However, no imaging modality could unequivocally identify constrictive physiology, possibly given the low fluid status caused by previous intravenous diuretic use. Moreover, while several RHC signs suggestive of constrictive pericarditis were present in our case, we were not able to assess the systolic area index, an additional sensitive marker for ventricular interdependence in constrictive pericarditis [1].

Diagnosing constrictive pericarditis is, therefore, challenging, particularly in those with other possible causes for RHF. Besides diagnosis, management could also be controversial. While findings of pericardial LGE at CMR could have led us to consider a trial of anti-inflammatory therapy before surgery [2], concomitant hemodynamically relevant left-to-right shunt possibly contributing to RV overload justified Heart-Teams’ decision to offer simultaneous pericardiectomy and shunt correction, also allowing us to reach the final histological diagnosis. Dense lymphoplasmacytic infiltrates and focal fibrosis with storiform pattern are key histopathological features of IgG4-related disease [3]. When present, IgG4 immunostaining is recommended to establish a definitive diagnosis.

IgG4-related disease is an autoimmune condition characterized by tissue IgG4 positive plasma cell infiltration [4–6]. Although cardiovascular involvement has been reported, constrictive pericarditis remains an unusual manifestation and often underrecognized presentation [6–8]. No guidelines exist regarding optimal treatment. Management usually relies on pericardiotomy and immunosuppressive therapies [7, 8].

Constrictive pericarditis remains a rare form of IgG4-related disease. A high-suspicion index, multimodality assessment and thorough investigation are critical in establishing the correct diagnosis.

## Supplementary Information

**Additional file 1.** Cardiac magnetic resonance (short axis, free-breathing cine) showing ventricular septum "d-shape".

## Data Availability

Not applicable.

## Abbreviations

CMR: Cardiac magnetic resonance
CT: Computed tomography
IgG4: Immunoglobulin-G4
LGE: Late-gadolinium enhancement
MI: Myocardial infarction
RHC: Right heart catheterization
RHF: Right heart failure
RV: Right ventricle
